# Exploring Cardiac Sympathetic Denervation as a Treatment Approach for Recurrent Ventricular Arrhythmias: A Concise Review

**Published:** 2024-03-14

**Authors:** Andrei Gurau, Frank Bosmans, Andreas Barth, Malcolm V. Brock, Jinny S. Ha

**Affiliations:** 1 Division of Thoracic Surgery, Johns Hopkins University School of Medicine, Baltimore, USA; 2 Division of Cardiology, Johns Hopkins University School of Medicine, Baltimore; 3 Department of Basic and Applied Medical Sciences, University of Ghent, Ghent, Belgium

**Keywords:** Sympathectomy, Surgical cardiac sympathetic denervation, Cardiac sympathetic denervation, Intractable ventricular tachyarrhythmias, Recurrent ventricular tachyarrhythmias, Refractory ventricular tachyarrhythmias, Non-ischemic cardiomyopathy, Ischemic cardiomyopathy, Implantable cardioverter defibrillator, Anti-arrhythmic medications, Catheter ablation, Autonomic nervous system, Sympathetic nervous system, Dysautonomia, Bilateral sympathectomy, Minimally invasive surgery, Robotic assisted thoracoscopic surgery, Video assisted thoracoscopic surgery, Minimally invasive cardiac arrhythmia surgery

## Abstract

Surgical Cardiac Sympathetic Denervation (CSD) has gained traction as a promising neuromodulatory therapy for Refractory Ventricular Tachyarrhythmias (RVT), particularly in patients with channelopathies and Ischemic (ICM) and Non-Ischemic Cardiomyopathies (NICM) who are refractory to conventional treatment. This mini review examines the pathophysiological role of the sympathetic nervous system in RVT and assesses the efficacy of Bilateral CSD (BCSD) through a literature review. Historical perspectives have traced the evolution of CSD from its initial use in intractable angina to its current application in ventricular arrhythmias. BCSD is associated with improved outcomes for refractory ventricular arrhythmias, with studies demonstrating approximately 60% reductions in implantable cardioverter defibrillator shocks and over 50% shock-and transplant-free survival at 1 year after BCSD. Notably, the 2017 AHA/ACC/HRS guidelines recommend Left CSD (LCSD) for certain etiologies of RVT, including congenital long QT syndrome, Catecholaminergic Polymorphic Ventricular Tachycardia (CPVT), and VT/VF storm. Both Video-Assisted Thoracoscopic Surgery (VATS) and Robot-Assisted Thoracoscopic Surgery (RATS) BCSD are performed, with shorter operative times for RATS. Yet, most RVT CSD studies have a small sample size; therefore, complications may be underreported because the studies are underpowered. Although BCSD has superior reported outcomes with respect to left CSD, there may be confounding factors due to the selection of healthier patients for BCSD. Additional comparative effectiveness and cost-effectiveness data are needed to guide clinical practice. In conclusion, BCSD can restore the quality of life of severely impacted RVT patients; however, the benefits must be weighed against procedure-related risks, and further research should clarify the impact on long-term morbidity and mortality.

## INTRODUCTION

The Autonomic Nervous System (ANS) is a key regulator of cardiovascular function, modulating the heart rate and rhythm, blood pressure, distribution of blood volume, and other essential life functions that ensure homeostasis. In addition to modulating heart rate and rhythm, the ANS is a key regulator of conduction, refractoriness, and susceptibility to arrhythmias [1]. Furthermore, sympathetic over activation is shown to be proarrhythmic, while a higher parasympathetic tone is protective. There is evidence showing the arrhythmogenic role of the sympathetic nervous system in Refractory Ventricular Tachyarrhythmias (RVT) in patients with channelopathies, and Ischemic (ICM) and Non-Ischemic Cardiomyopathies (NICM) [2]. Despite aggressive medical management with anti-arrhythmic medications and catheter ablations, patients with RVT continue to have breakthrough tachyarrhythmias requiring Implantable Cardioverter Defibrillator (ICD) shocks. Beyond the higher mortality and morbidity risk secondary to the underlying disease, RVT patients who are experiencing multiple ICD shocks find their quality of life profoundly and deleteriously impacted [3]. There has been great interest in finding an effective therapy for RVT. ANS neuromodulation has emerged as a promising therapeutic option, with various therapies seeking to either increase parasympathetic tone or decrease sympathetic tone. Surgical Cardiac Sympathetic Denervation (CSD) is the most decisive neuromodulatory therapy removing sympathetic stimulation to the heart, by transecting sympathetic nerve fibers prior to innervating the heart [4]. We now turn to a detailed examination of the existing literature to further understand which patients will benefit most from cardiac sympathetic denervation for RVT.

## LITERATURE REVIEW

### Historical perspective

The first documented cardiac sympathetic denervation was performed in 1916 by the pioneering surgeon and anatomist, Thoma Ionescu, in Bucharest, Romania [5]. The indication for this treatment was intractable angina pectoris. Reportedly, Ionescu noted that arrythmias improved as well [6]. After dissemination of the case-report, CSD gained traction as an acceptable treatment for intractable angina pectoris until beta-blocker medications became widely available [6]. In the late 1920s through 1960s there were reports of CSD for treatment of supraventricular tachyarrhythmias; in the early 1960s Estes and Izlar published their case-report on the use of bilateral CSD for treatment of recurrent ventricular tachycardia and noted prior case reports and series that were the basis of their work [7]. During the 1970s, the efficacy of CSD was further studied and established in patients with hereditary channelopathies and has since gone on to be studied in patients with ventricular tachyarrhythmias due to structural heart disease [6]. To date, the resulting data has been promising, with the 2017 AHA/ACC/HRS guidance on ventricular arrhythmia management recommending LCSD for several RVT syndromes [8].

### CSD indication and patient selection

CSD is indicated in patients with ventricular tachycardia or ventricular fibrillation when conventional treatments, including antiarrhythmic drugs and catheter ablation fail to control refractory ventricular tachyarrhythmias. The procedure is often considered in patients who experience multiple ICD shocks. The benefit of CSD for individuals with advanced heart failure and slow monomorphic VT from scar tissue may be limited, as indicated by prior reports [9,10]. Current contraindications to CSD include advanced heart failure (NYHA class IV due to concerns for persistent postoperative hypotension) and severe lung disease.

### CSD outcomes

Commonly reported outcomes of CSD are metrics of mortality and reduction in arrhythmia burden; however, surrogate outcomes for these measures are often used instead. The frequently reported surrogate is typically a reduction in ICD shocks with respect to the number of shocks before surgery. Most studies typically report a reduction in ICD shocks in over 50%−60% of patients who underwent CSD. In an expansive systematic review and meta-analysis, Chihara, et al., aggregated data and examined pooled RVT outcomes after CSD. The authors showed that across various etiologies for RVT, patients that underwent CSD had a pooled ICD shock-free rate of about 60%, over a 50% ICD shock-and transplant-free survival, and a 29.5% rate of mortality and transplantation. CSD was associated with the reduction of ventricular arrhythmias, improved survival without the need for transplants, and a moderate impact on overall mortality and transplantation rates [11].

### Operative considerations

#### Left-sided unilateral *vs*. bilateral sympathectomy:

While Left CSD (LCSD) has traditionally been performed, Bilateral CSD (BCSD) has been shown to offer a better response to surgery. Several notable studies from Vaseghi, et al., showed that BCSD was associated with a more durable ICD-shock-free and cardiac-transplant-free survival than LCSD at one year post-operative follow-up [9]. As a limitation to interpreting these results, these were retrospective studies with more patients undergoing BCSD. Furthermore, those who underwent LCSD, might have only tolerated general anesthesia long enough to undergo the first half of the operation (LCSD only), which may be a confounding factor [9]. However, while the complication rates were similar in terms of ICD shock-free and transplant-free survival between the LCSD and BCSD groups, LCSD was associated with an approximately 15% higher mortality and progression to transplant (p=0.042) in a systematic meta-analysis [11].

#### Operative approaches, technique, and associated comparative outcomes:

CSD is performed using a Minimally Invasive Surgical approach (MIS) [11]. MIS can be Video-Assisted Thoracoscopic Surgery (VATS) or Robot-Assisted Thoracoscopic Surgery (RATS). Melinosky, et al., compared the clinical outcomes between VATS and RATS CSD in 77 patients. This study showed that both groups had a significant decrease in ICD shocks at one-year post-operation with respect to preoperative ICD shocks (p<0.001). Additionally, there was no difference in the number of shocks at one-year post-operation between the two groups (p=0.75), nor was there a difference in the median postoperative length of stay (three days, p=0.21). However, RATS CSD was a median of 20 minutes faster and associated with fewer pneumothoraces than VATS CSD (p=0.022 and p=0.004, respectively) [4]. Yet, VATS CSD is far more commonly performed than RATS CSD, but with the ongoing increase in utilization of robotic-assisted surgery, this may change in the near future [12]. Single lung ventilation is typically achieved with the placement of a double lumen endotracheal tube. Given that left CSD is performed first, patients are typically first positioned in the right lateral decubitus position to start the operation, and then rotated to the left lateral decubitus position to complete the BCSD. For VATS, ports can be placed in the 3^rd^ and 7^th^ intercostal spaces on the midaxillary line, with the 3^rd^ space as the working port. For RATS, working ports are placed in the 4^th^ and 8^th^ intercostal spaces, with the camera placed in the 6^th^ interspace port. Surgical techniques typically involve removal of the inferior aspect of the stellate ganglion, followed by resection of the T2 thoracic through the T4 paravertebral ganglia, with the fourth rib serving as the respective landmark [4,11]. One common variation in the surgical technique is the additional resection of Kuntz’s Nerve (KN). KN is thought to possess sympathetic fibers that may otherwise escape resection, thereby reducing the efficacy of the operation. Complicating this matter in most cases is the challenge to identify KN. Marhold, et al., explored this question in a cadaveric study (n=33), in which the authors showed that KN was identifiable in approximately 12% of cadavers during VATS. In contrast, KN was identifiable in 66% after anatomical dissection [13]. Current data suggest that KN resection is not necessary for a durable response to the operation, with pooled data comparing BCSD with KN resection to BCSD alone specifically showed that there was no statistical difference in ICD-shock, transplant, and mortality free survival between those two groups [11]. In summary to achieve CSD, VATS BCSD with inferior stellectomy with T2 through T4 paravertebral ganglionectomy is the most commonly performed procedure to achieve CSD and the associated clinical benefits.

#### Post-operative complications:

Pooled data from 254 patients that underwent surgical cardiac sympathetic denervation revealed the following top four complications: Pneumothorax (5.5%), Horner’s syndrome (4.3%), hemothorax (1.97%), and azygos vein/venous plexus injury (0.79%) [11]. Another well recognized complication of CSD is compensatory hyperhidrosis, Chihara, et al., reported an occurrence of this complication in 0.39% of the pooled data [11]. However, this may differ across centers, with another study reporting compensatory changes in sweating in 10% of patients that underwent CSD [10]. At our center, we counsel anyone with preoperative primary focal hyperhidrosis (PFH: palmar, facial, or axillary) who needs a CSD that they most likely have a high risk of having compensatory sweating postoperatively. In our experience, thoracic sympathetic denervation in PFH results in the majority of these patients having compensatory sweating changes [14].

## DISCUSSION

The literature presents compelling evidence of the utility of BCSD as an effective and minimally invasive therapy for RVT. Multiple studies have shown significant reductions in the burden of ICD shocks and improvements in transplant-free survival after BCSD. As such, the 2017 AHA/ACC/HRS guidelines recommend BCSD for several treatments including refractory ventricular arrhythmia syndromes [8].

Several notable findings emerge from the review of the literature. First, BCSD seems to confer better outcomes than left-sided CSD alone in retrospective analyses, with an approximately 15% lower risk of mortality and transplantation. Notably, while these non-randomized studies had baseline confounders between groups, similar overall complication rates and operative durations support a broader adoption of the bilateral approach. However, given the heterogeneity and small sample sizes, it is possible that some complications are underreported due to the underlying study design parameters, reporting limitations, or type II error. Second, while VATS is still more prevalent for CSD, RATS CSD shows clear advantages with shorter operative times and fewer residual pneumothoraces, as demonstrated in one of the largest single-center retrospective studies published [4]. Finally, procedure-related complications highlight the necessity of appropriate patient selection and counseling regarding risks *versus* debilitating symptoms from RVT and decision to perform LCSD *versus* BCSD.

Despite the accumulating literature, knowledge gaps persist and require further research. Long-term follow-up data are limited, with most studies reporting only the short-term frequency of arrhythmia after BCSD. Additionally, well-designed, and adequately powered prospective studies directly comparing VATS, RATS, and unilateral and bilateral approaches would better guide evolving clinical practice. A planned “Left Cardiac Sympathetic Denervation (LCSD) for cardiomyopathy feasibility pilot study” was suspended in part due to the COVID-19 pandemic. Fortunately, “Cardiac Sympathetic Denervation (CSD) for Prevention of Ventricular Tachyarrhythmias (PREVENT VT)”, will examine outcomes after BCSD in 40 patients and is estimated to be completed in August of 2024 [15,16]. Other areas for future study include of cost-effectiveness evaluations and standardization of key metrics for long-term outcome reporting. Finally, insights into how CSD works at a molecular physiological level are lacking. One possible explanation could be related to dysfunction of ion channels expressed in the ANS, in which the biophysical consequences of channelopathies could alter membrane excitability to increase the sympathetic tone or decrease parasympathetic activity, thereby affecting cardiac function [17]. Indeed, the ANS enervates every organ in the human body and dysregulation may not only affect the heart but can also lead to, among others, PFH, severe anxiety and depression, migraine-type headaches, chronic fatigue, irritable bowel syndrome, attention deficit disorder, and insomnia [18]. Given the autonomic origin of cardiac sympathetic activation, and the observation that denervation reduces sympathetic tone, it is possible that functional consequences of channelopathies, such as mutations in voltage-gated Na^+^ channels that initiate action potentials, are exerted *via* the sympathetic nerve and can be arrhythmogenic in patients with structural heart disease [19]. A confounding factor worth noting here is that brain regions that govern the ANS as well as cardiomyocytes themselves also employ ion channels to drive electrical signaling [20].

## CONCLUSION

In conclusion, CSD has emerged as a viable option for reducing the RVT burden, but practicing clinicians must judiciously weigh the benefits against established procedure-related risks. Ongoing mechanistic, operative, and comparative effectiveness investigations will refine future patient selection and the choice between surgical techniques. For severely impacted individuals, CSD holds promise for improving quality of life and potentially long-term survival.
